# Coat protein of rice stripe virus enhances autophagy activity through interaction with cytosolic glyceraldehyde-3-phosphate dehydrogenases, a negative regulator of plant autophagy

**DOI:** 10.1007/s44154-023-00084-3

**Published:** 2023-03-23

**Authors:** Wanying Zhao, Li Wang, Lipeng Li, Tong Zhou, Fei Yan, Heng Zhang, Ying Zhu, Ida Bagus Andika, Liying Sun

**Affiliations:** 1 State Key Laboratory of Crop Stress Biology for Arid Areas, College of Plant Protection, Northwest A&F University, Yangling, 712100 Shaanxi China; 2 Institute of Plant Protection, Jiangsu Academy of Agricultural Sciences, Nanjing, 210095 China; 3 Institute of Plant Virology, Ningbo University, Ningbo, 312362 China; 4 State Key Laboratory for Managing Biotic and Chemical Threats to the Quality and Safety of Agro-Products, Institute of Virology and Biotechnology, Zhejiang Academy of Agricultural Sciences, Hangzhou, 310021 China; 5 College of Plant Health and Medicine, Qingdao Agricultural University, Qingdao, 266109 China

**Keywords:** RSV, Coat protein, Autophagy, GAPC, ATG3

## Abstract

Viral infection commonly induces autophagy, leading to antiviral responses or conversely, promoting viral infection or replication. In this study, using the experimental plant *Nicotiana benthamiana*, we demonstrated that the rice stripe virus (RSV) coat protein (CP) enhanced autophagic activity through interaction with cytosolic glyceraldehyde-3-phosphate dehydrogenase 2 (GAPC2), a negative regulator of plant autophagy that binds to an autophagy key factor, autophagy-related protein 3 (ATG3). Competitive pull-down and co-immunoprecipitation (Co-IP)assays showed that RSV CP activated autophagy by disrupting the interaction between GAPC2 and ATG3. An RSV CP mutant that was unable to bind GAPC2 failed to disrupt the interaction between GAPC2 and ATG3 and therefore lost its ability to induce autophagy. RSV CP enhanced the autophagic degradation of a viral movement protein (MP) encoded by a heterologous virus, citrus leaf blotch virus (CLBV). However, the autophagic degradation of RSV-encoded MP and RNA-silencing suppressor (NS3) proteins was inhibited in the presence of CP, suggesting that RSV CP can protect MP and NS3 against autophagic degradation. Moreover, in the presence of MP, RSV CP could induce the autophagic degradation of a remorin protein (NbREM1), which negatively regulates RSV infection through the inhibition of viral cell-to-cell movement. Overall, our results suggest that RSV CP induces a selective autophagy to suppress the antiviral factors while protecting RSV-encoded viral proteins against autophagic degradation through an as-yet-unknown mechanism. This study showed that RSV CP plays dual roles in the autophagy-related interaction between plants and viruses.

**Supplementary Information:**

The online version contains supplementary material available at 10.1007/s44154-023-00084-3.

## Introduction

Autophagy is an essential and conserved process that leads to the degradation of intracellular components, including soluble proteins, misfolded proteins, organelles, and macromolecular complexes (Yu et al. 2018). It is crucial for cell homeostasis maintenance, growth and development, and environmental stress responses (Prasanth et al. 2011)and (Choi et al. 2013). According to the mechanism of action, there are three types of autophagy: macroautophagy, microautophagy, and chaperone-mediated autophagy (CMA). Only macroautophagy and microautophagy have been described in plants, and there is no evidence of CMA in plants (Yang and Liu 2022). Macroautophagy (hereafter referred to as autophagy) is the most common type of autophagy and has been extensively studied (Li and Vierstra 2012). In brief, autophagy is a catabolic process in which substrates are sequestered within double-membraned vesicles termed autophagosomes. The mature autophagosomes are then delivered to vacuoles or lysosomes for degradation, and the vehicles are released back into the cytosol for recycling. The complex series of processes underlying autophagosome initiation and maturation depends on the coordinated action of a conserved set of autophagy-related (ATG) proteins (Mizushima et al. 2011; Rubinsztein et al. 2012). ATG8/LC3 (autophagy-related protein 3/light chain 3) family proteins have emerged as central players in autophagosome biogenesis and cargo recruitment (Iman et al. 2017; Slobodkin and Elazar 2013). ATG8 lipidation is mediated by two ubiquitin-like conjugation pathways involving the E1-like ligase ATG7, the E2-ligase ATG3, and the E3-like ligase ATG5-ATG12-ATG16 complex (Yu et al. 2018). Cytosolic glyceraldehyde-3-phosphate dehydrogenases (GAPCs), which serve as negative regulators, interact with ATG3 to suppress autophagy (Han et al. 2015).

Recent studies reveal that autophagy, as an essential physiological process, participates in a variety of stress responses, including nutrient deprivation and immune activation (Avin-Wittenberg 2019; Chen et al. 2021). When suffering from nutritional starvation, plants enhance autophagic activity by promoting the expression of autophagy genes, boosting the metabolism and circulation of nutrients, and ensuring their survival (Masclaux-Daubresse et al. 2017). Moreover, autophagy plays a vital role in the interaction between plants and pathogens. However, its roles are complex and diverse in that autophagy can either enhance or inhibit plant defense responses (Leary et al. 2018; Gallegos 2018). Studies have shown that autophagy is activated in response to various DNA and RNA viruses with negative consequences for virus accumulation, suggesting the integration of autophagic mechanisms in basal antiviral defenses (Hafrén et al. 2017; Haxim et al. 2017; Li et al. 2018). Many viral proteins are targeted by the autophagy machinery for degradation. For example, viral silencing suppressors, such as HC-Pro encoded by tobacco etch virus and 2b encoded by cucumber mosaic virus, are degraded by autophagy, resulting in the suppression of virus accumulation (Nakahara et al. 2012; Jeon et al. 2017). Moreover, the RNA-dependent RNA polymerase (RdRp) of turnip mosaic virus (TuMV) is degraded by the autophagy pathway via direct interaction with ATG6/Beclin1, which is proposed to act as a cargo receptor (Li et al. 2018). Some other viral proteins are also subjected to autophagic degradation, such as the virulence-associated protein βC1 from cotton leaf curl Multan virus (CLCuMuV) (Haxim et al. 2017) and movement protein (MP) of citrus leaf blotch virus (CLBV) (Niu et al. 2021). On the other hand, viruses have also developed machinery to suppress autophagic activity to conquer the antiviral response. For instance, barley stripe mosaic virus (BSMV) subverts antiviral autophagy with the help of the γb protein, which disrupts ATG7-ATG8 interaction and thus impairs autophagosome formation through ATG8 binding (Yang et al. 2018). The coat protein of Chinese wheat mosaic virus (CWMV) inhibits autophagy through the upregulation of and interaction with GAPCs (Niu et al. 2022). P38 encoded by turnip crinkle virus inhibits autophagy by directly sequestering ATG8 proteins (Shukla et al. 2021).

Rice stripe virus (RSV), a type species of the genus *Tenuivirus*, often causes enormous losses in the production and quality of rice crops globally (Kyong et al. 2013). RSV is transmitted by an insect vector, the small brown planthopper (*Laodelphax striatellus Fallén*) in a persistent propagative manner (Falk and Tsai 1998; Heydarnejad et al. 2006; Huo et al. 2014). The RSV genome contains four negative single-stranded RNA (–ssRNA) segments (Falk and Tsai 1998; Liu et al. 2018). The RNA1 is negative sense and encodes the RdRp (RNA-dependent RNA polymerase) (Barbier et al. 1992). The RNA2 to RNA4 strands are ambisense and encode two open reading frames (ORFs) in opposite orientations on the viral RNA (vRNA) and viral complementary RNA (vcRNA). RSV vRNA2 encodes the NS2 protein that functions in RNA silencing suppression, and vcRNA2 encodes a glycoprotein precursor (NSvc2) (Zhenguo et al. 2011). RSV vRNA3 encodes a non-structural protein (NS3) as a viral RNA suppressor (VSR), and vcRNA3 encodes a nucleocapsid protein that functions as a coat protein (CP), encapsidating the viral genome RNAs (Lian et al. 2014; Kim et al. 2017). RSV vRNA4 encodes a major nonstructural disease-specific S-protein (SP), and vcRNA4 encodes an MP (Kakutani et al. 1990; Xiong et al. 2008). Two structural proteins, RdRp and CP, form filamentous virions that are also referred to as ribonucleoprotein particles (RNPs). A previous study revealed that RSV NS3 triggers autophagy by eliciting the unfolded protein response (UPR) in *Nicotiana benthamiana* (an experimental host of RSV). A potential selective autophagy receptor, phosphatidylinositol 2-monophosphate (P3IP) from the *N. benthamiana* plant was found to interact with RSV NS3 and mediate its degradation to suppress viral replication (Jiang et al. 2021). Recently, a study indicated that RSV MP facilitates the autophagic degradation of the remorin protein (NbREM1), which mediates the inhibition of viral cell-to-cell movement (Fu et al. 2018). These studies demonstrated that autophagy is highly implicated in plant defense and RSV infection.

To understand the mechanism by which RSV induces autophagy, we examined the effect of RSV protein expression on the induction of autophagy. We found that three RSV proteins, namely CP, MP, and SP, could activate autophagy. Hence, we focused on RSV CP, the most abundant viral protein, to decipher the mechanism of autophagy induction via the host components involved in autophagy activation.

## Results

### RSV CP induces autophagy

**Fig. 1:**
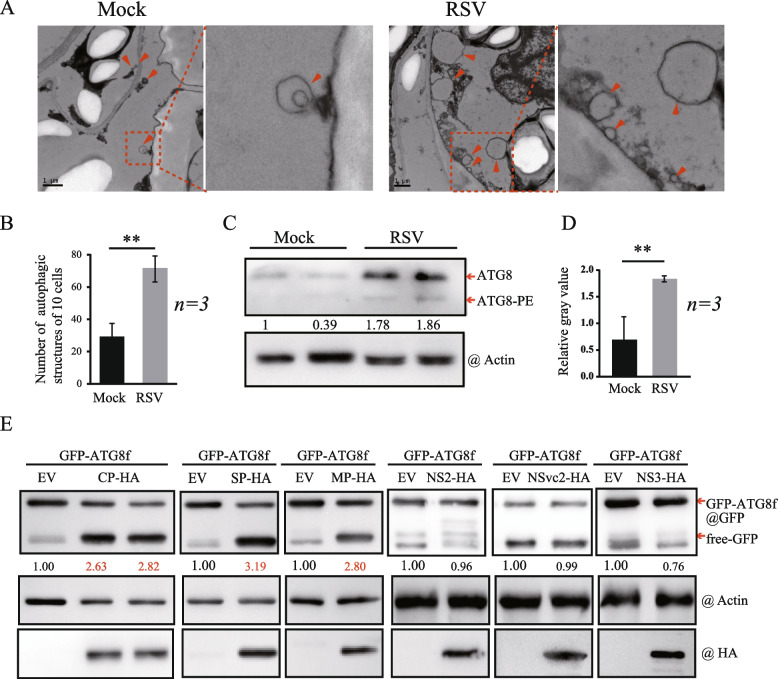
RSV and RSV coding proteins induce autophagy. **A** Representative transmission electron microscope (TEM) images of *N. benthamiana* leaf cells with (right picture) or without (left picture) RSV infection. The ultrastructure of autophagic bodies was observed in the cells (indicated by arrows). **B** Quantification of the number of autophagosomes in the cells of leaves described in (A). Each bar represents the total number of autophagic bodies from 10 cells obtained from three independent experiments. “**” indicates *P* < 0.01 (Student’s *t*-test). **C** Western blotting analyses of the accumulation of ATG8 and its lipidated form (ATG8-PE) in RSV-infected *N. benthamiana* plants. Total protein samples were extracted from the upper leaves of plants and subjected to immunoblotting analysis with anti-ATG8 and anti-actin antibodies. **D** Quantification of the relative autophagic activity in (C). Gray value statistics were normalized to the internal control actin and control plant values, which were set to 1.0. Values represent the standard deviation obtained from three independent experiments. “*” and “**” indicate *P* < 0.05 and *P* < 0.01, respectively (Student’s *t*-test). **E** Western blotting analysis of GFP accumulation in *N. benthamiana* leaves co-expressing RSV proteins CP-HA, SP-HA, MP-HA, NS2-HA, NSvc2-HA, and NS3-HA with GFP-ATG8f. The accumulation of GFP-ATG8f and free GFP was detected with an anti-GFP antibody, and RSV coding proteins were detected via their fused tag using an anti-HA antibody. Sample loading was normalized to the endogenous actin protein using an anti-actin antibody

A previous study showed that RSV and RSV-encoded proteins induce autophagy in the *N. benthamiana* plant (Jiang et al. 2021). In the present study, we also found that RSV infection triggered a high level of autophagic activity. Using TEM, we observed that the number of autophagosomes was increased in RSV-infected plants compared with uninfected plants (Fig. 1A and B). The accumulation of ATG8 conjugated to phosphatidylethanolamine lipids (ATG8-PE) reflects the autophagic activity in the cell (Kabeya et al. 2000). The western blot analysis revealed the presence of ATG8-PE, represented by a faster-migrating band, in RSV-infected plants, whereas the ATG8-PE band was hardly detected in uninfected plants (Fig. 1C and D), suggesting that RSV infection triggered autophagy.

To investigate the role of RSV proteins in the induction of autophagy, we transiently expressed all RSV-encoded proteins except for RdRP in the epidermal cells of *N. benthamiana* leaves using agroinfiltration and evaluated their ability to induce autophagy. Since GFP-tagged ATG8 has been widely used for monitoring autophagosome formation and autophagic flux, GFP-ATG8 (ATG8f) was used to examine the effect of viral proteins on autophagic activity in *N. benthamiana*. Each viral protein was then co-expressed with GFP-ATG8f to monitor the accumulation of cleaved GFP (free GFP), which is a consequence of autophagy (Adachi et al. 2017). The autophagic flux was represented by the ratio of the amount of free GFP (around 27 KDa) to fused GFP-ATG8f in the lane. As shown in Fig. 1E, two bands were detected on the immunoblots (upper panel), and the cleaved GFP band is indicated with an arrow (Fig. 1E). The protein accumulation was normalized with the amount of actin protein. The ratio reflecting autophagic flux was calculated and is listed at the bottom of the western blot panel (Fig. 1E). Compared to the absence of viral protein expression, co-expression with CP, SP, and MP increased the accumulation of free GFP by around twofold, whereas NS2, NSvc2, and NS3 expression did not elevate the ratio of autophagic flux (Fig. 1E). These results indicated that RSV CP, SP, and MP likely induce autophagy when expressed alone in *N. benthamiana* cells.

### RSV CP interacts with GAPCs

**Fig. 2:**
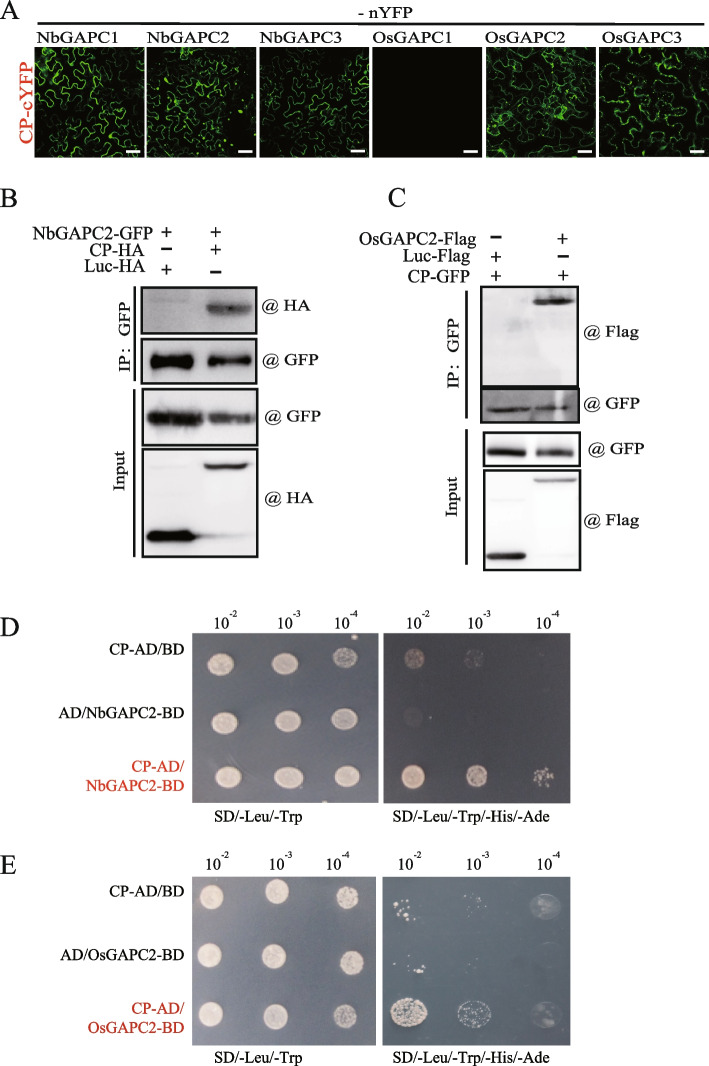
RSV CP interacts with GAPCs. **A** BiFC assays to examine the interaction between RSV CP and GAPCs. RSV CP and GAPCs (NbGAPC1, NbGAPC2, NbGAPC3, OsGAPC1, OsGAPC2, and OsGAPC3) were fused to the N-terminal or C-terminal portions of the split yellow fluorescent protein (nYFP or cYFP, respectively) and transiently co-expressed in *N. benthamiana*. The reconstituted YFP fluorescence in epidermal cells was observed by confocal laser scanning microscopy. Scale bars, 20 μm. **B** and **C** In vivo co-immunoprecipitation assay to examine the interaction of CP with NbGAPC2 or OsGAPC2. RSV CP fused to an HA tag or GFP, NbGAPC2 fused to GFP, and OsGAPC2 fused to Flag were transiently expressed in *N. benthamiana* leaves, and immunoprecipitations were performed with an anti-GFP antibody. Protein samples before and after immunoprecipitation were analyzed by western blot with anti-HA, anti-Flag, and anti-GFP antibodies. **D** and **E** Yeast two-hybrid analysis of the interaction between CP and NbGAPC2 or OsGAPC2. The CP and NbGAPC2 or OsGAPC2 were inserted into pGADT7(AD) and pGBKT7(BD) plasmids. The combination plasmids were co-introduced into the yeast AH109 strain and cultured on a selective medium lacking SD-Leu-Trp and SD-Leu-Trp-His-Ade for 3–5 days

Because CP is the most abundant viral protein in the host cells during viral infection, we next focused on deciphering the mechanism underlying the induction of autophagy by RSV CP. To identify the host factor that might be associated with the induction of autophagy by CP, we carried out a Co-IP (Co-Immunoprecipitation) assay using RSV CP as a bait protein and used LC–MS/MS to analyze the protein interactors. For the preparation of the bait protein, GFP was fused to the C-terminal of RSV CP and expressed in *N. benthamiana* leaf epidermal cells using agroinfiltration. The total proteins were extracted and incubated with the covalently coupled GFP beads to enrich the bait protein and its interactors. The leaves expressing non-fused GFP protein were treated in parallel as a control (Supplementary Fig. 1A). The proteins were eluted and separated via SDS-PAGE. The bands corresponding to bait proteins were shown in a bulk accumulation, while the additional bands in the CP-GFP samples were selected for analysis by LC–MS/MS (Supplementary Fig. 1A, arrowhead). LC–MS/MS and bioinformatics analyses for protein identification revealed that GAPC2, a regulator of autophagy, might interact with CP (Supplementary Fig. 1B). *N. benthamiana* and *Oryza sativa* encode three GAPC proteins (GenBank accession numbers are listed in Supplementary Table 1). First, we used BiFC (Bimolecular Fluorescence Complementation) assays for the visualization of protein interactions in living cells. The complete coding sequences (cDNA) of NbGAPCs and OsGAPCs were cloned and fused to the N-terminal part of the split YFP (nYFP), while the RSV CP was fused to the C-terminal region of the split YFP (cYFP). The combinations of split YFP proteins were co-expressed, and the reconstituted fluorescence, indicating protein interaction, was observed via confocal laser scanning microscopy (CLSM). As shown in Fig. 2A, YFP fluorescence was observed when RSV CP and NbGAPCs were co-expressed. In contrast, no fluorescence signals were observed in the non-fused control, suggesting that RSV CP interacts with all NbGAPCs (NbGAPC1, NbGAPC2, and NbGAPC3) and two OsGAPCs (OsGAPC2 and OsGAPC3; Fig. 2A).

To confirm the interaction of CP with GAPCs, we performed a Co-IP assay of RSV CP and NbGAPC2 (Fig. 2B) or OsGAPC2 (Fig. 2C) co-expressed in *N. benthamiana.* A luciferase gene (Luc) was used as a control. The detection of the co-immunoprecipitated proteins revealed that CP could bind to NbGAPC2 and OsGAPC2 but not to Luc (Fig. 2B and C). We also carried out a yeast two-hybrid assay to further confirm the interaction of CP with GAPCs (Fig. 2D and E). For the yeast two-hybrid assay, NbGAPC2 and OsGAPC2 were fused to the DNA-binding domain and RSV CP was fused to the activation domain of Gal4. AH109 yeast cells containing the combination plasmids were cultured at different dilutions on non-selective (-Leu-Trp) and selective (-Leu-Trp-His-Ade) plates. The results showed that CP interacted with either NbGAPC2 or OsGAPC2 (Fig. 2D and E). Together, BiFC, Co-IP, and yeast two-hybrid assays strongly indicated the presence of interactions between RSV CP and GAPCs.

### The amino acid at position four in RSV CP is essential for binding with GAPC2

**Fig. 3:**
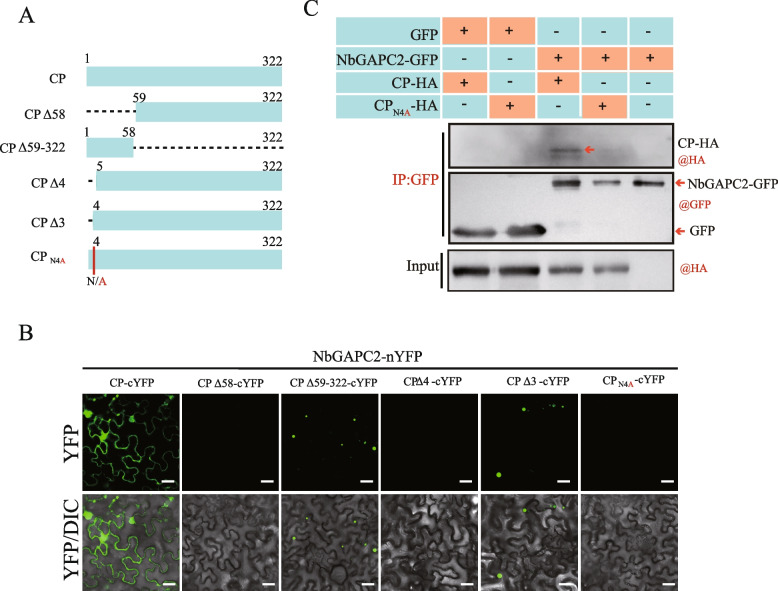
The fourth amino acid (Asn) of CP is essential for binding to NbGAPC2. **A** Schematic representation of the CP mutants analyzed in this study. The amino acid positions of deletions and point mutations in the CP sequence are shown above the diagram. N/A indicates that Asn (N) was replaced by Ala (A). **B** BiFC assay to examine the interaction of NbGAPC2 and CP or CP mutants. NbGAPC2 and CP or CP mutants (CPΔ58, CPΔ59-322, CPΔ4, CPΔ3, and CPN4A) were fused to the N-terminal or C-terminal portions of the split yellow fluorescent protein nYFP or cYFPnd transiently co-expressed in *N. benthamiana*. The reconstituted YFP fluorescence in epidermal cells were observed by confocal laser scanning microscopy. Scale bars, 20 μm. **C** In vivo co-immunoprecipitation assay to examine the interaction of NbGAPC2 with CP or CP_N4A_. CP and CP_N4A_ fused to an HA tag and NbGAPC2 fused to GFP were transiently expressed in *N. benthamiana* leaves, and immunoprecipitations were performed with an anti-GFP antibody. Protein samples before and after immunoprecipitation were analyzed by western blot with anti-HA and anti-GFP antibodies

We further mapped the regions in RSV CP that are essential for its interaction with NbGAPC2. A series of truncated CP proteins were fused to the N-terminal part of YFP, and a BiFC assay was performed. The results showed that the N-terminal region of CP (1–58 amino acids) was responsible for its interaction with NbGAPC2 (Fig. 3A and B). Interestingly, the interaction was observed when the three amino acids at the N-terminal of CP were deleted but not observed when the fourth amino acid (asparagine) at the N-terminal was also deleted, suggesting that the fourth amino acid was essential for binding to GAPC2 (Fig. 3A and B). A CP mutant with the substitution of asparagine (N) at the fourth amino acid position with alanine (A; CP_N4A_) was further examined for its interaction with GAPC2 by BiFC and Co-IP assays. The results showed that the CP_N4A_ mutant failed to interact with NbGAPC2 (Fig. 3B and C) and OsGAPC2 (Supplementary Fig. 2).

**Fig. 4:**
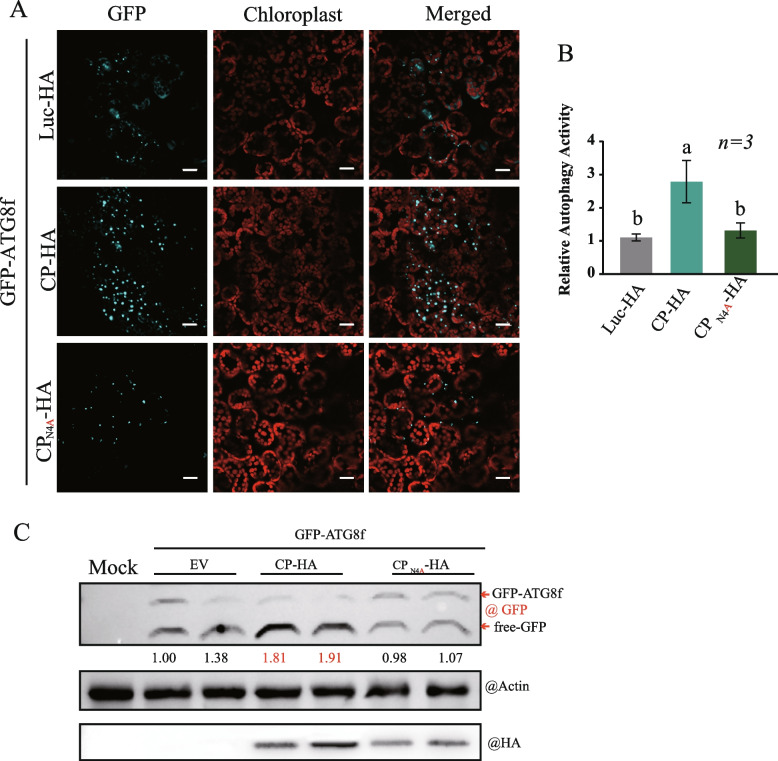
Activation of autophagy by transiently expressed RSV CP. **A** The autophagy activity of *N. benthamiana* leaves expressing CP or CP_N4A_ was assessed by the autophagy marker GFP-NbATG8f. Luc-HA, CP-HA, or CPN4A-HA were co-expressed with GFP-ATG8f in *N. benthamiana* using *Agrobacterium* infiltration. The GFP fluorescence in epidermal cells was observed by confocal laser scanning microscopy. GFP-NbATG8f fusion proteins are in cyan, and chloroplasts are in red. Scale bars, 20 μm. **B** The quantification of the numbers of autophagic structures in the cells of leaves described in (A). More than 100 mesophyll cells for each treatment were used for the quantification. The relative autophagic activity in Luc-HA-infected plants was normalized to control plant values, which were set to 1.00. Values represent the means from three independent experiments. The significant differences are marked with different lowercase letters over the columns (P < 0.05, one-way ANOVA). **C** The protein samples obtained from (A) were subjected to western blot analysis using anti-GFP, anti-HA, and anti-actin antibodies. The accumulation of GFP was normalized to actin. The ratio of free GFP via total GFP (GFP-ATG8f plus free-GFP) was compared to the control sample (EV), which was set to 1.00

To determine whether CP_N4A_ maintained the ability to induce autophagy, we co-expressed CP or the CP_N4A_ mutant with GFP-tagged ATG8f for monitoring autophagosome formation and autophagic flux. The proteins were transiently expressed by agroinfiltration in *N. benthamiana* following treatment with the cysteine protease inhibitor aloxistatin (E64d). As shown in Fig. 4, GFP-NbATG8f, an autophagosome marker, was observed as punctate autophagic bodies by CLSM. The results revealed an increased number of autophagic bodies in the presence of wild-type CP but not the CP_N4A_ mutant compared with the Luc protein, which was used as a control (Fig. 4A and B), indicating that the CP_N4A_ mutant had no ability to induce autophagy. Moreover, the total proteins were extracted from the plant tissue used for observing autophagic bodies (Fig. 4A) and subjected to the detection of GFP accumulation. The ratio of the amount of cleaved GFP to GFP-ATG8f, reflecting the autophagic flux, was calculated (Fig. 4C). Consistently, the ratio representing autophagic flux was remarkably increased in the presence of wild-type CP but not the CP_N4A_ mutant. These results suggested that RSV CP regulates autophagy through its interaction with GAPCs and the fourth amino acid at the CP N-terminal is crucial for its interaction with NbGAPC2. The CP_N4A_ mutant failed to interact with GAPC2 activate autophagy.

### RSV CP disrupts the interaction of GAPC2 and ATG3

**Fig. 5:**
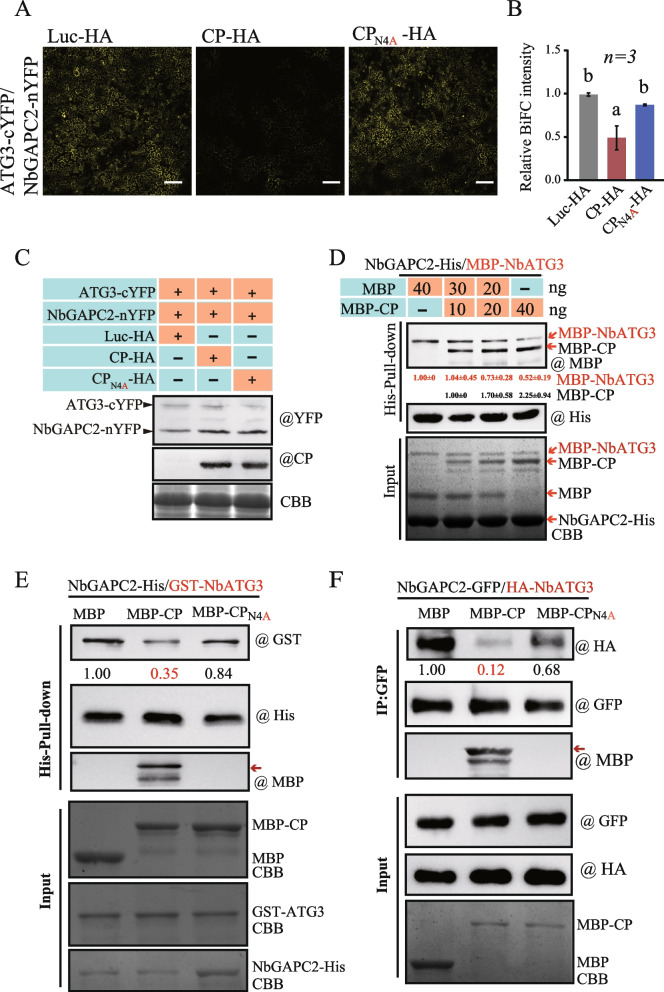
RSV CP disrupts the interaction between NbGAPC2 and NbATG3. **A **The interaction of NbGAPC2 and NbATG3 was evaluated by BiFC assay. The recombinant plasmids of NbGAPC2-nYFP and NbATG3-cYFP were co-expressed with CP-HA or CPN4A-HA in *N. benthamiana *leaves. Luc-HA was used as the control. The reconstituted YFP fluorescence in epidermal cells were observed by confocal laser scanning microscopy. Scale bars, 20 μm. **B **The intensity of reconstituted YFP fluorescence (BiFC) described in (A) was normalized to the control sample (Luc-HA). The error bar represents the mean intensity of YFP fluorescence quantified by ImageJ from 30 pictures obtained from three independent experiments. Different letters above the bars indicate significant differences (*P* < 0.05, one-way ANOVA). **C **The protein accumulation of experiment described in (A) examined by western blot using GFP and CP antibodies. **D **Competitive pull-down assay. NbGAPC2-His, MBP-NbATG3, MBP, MBP-CP were expressed and purified from *E. coli *BL21(DE3). An equivalent amount (20 μg) of NbGAPC2-His and MBP-NbATG3 was incubated with MBP (40, 30, 10, 0 ng), MBP-CP (0, 10 30 , 40 ng) at 4 ℃ for 1 hour. A pull-down assay was performed with His-specific affinity resin (Ni-NTA agarose) and analyzed by western blotting using His, and MBP antibodies. The accumulation of MBP-NbATG3 protein was quantified by Image J and set to 1.00 for the control sample (MBP). The number indicated the relative MBP-NbATG3 accumulation, which was normalized to NbGAPC2-His and compared to the control sample. **E **Competitive pull-down assay. NbGAPC2-His, GST-NbATG3, MBP, MBP-CP, and MBP-CPN4A were expressed and purified from *E. coli *BL21(DE3). An equivalent amount (20 μg) of NbGAPC2-His and GST-NbATG3 was incubated with MBP, MBP-CP, or MBP-CPN4A protein at 4 ℃ for 1 hour. A pull-down assay was performed with His-specific affinity resin (Ni-NTA agarose) and analyzed by immunoprecipitation with anti-GST, anti-His, and anti-MBP antibodies. The accumulation of GST-NbATG3 protein was quantified by ImageJ and set to 1.00 for the control sample (MBP). The number indicated the relative GST-NbATG3 accumulation, which was normalized to NbGAPC2-His and compared to the control sample. **F **Competitive Co-IP assay. NbGAPC2-GFP and HA-NbATG3 were co-expressed in *N. benthamiana *leaves and immunoprecipitated with anti-GFP beads incubated with purified MBP, MBP-CP, MBP-CPN4A proteins at 4 ℃. Input and IP proteins were analyzed by western blot with anti-HA, anti-GFP, and anti-MBP antibodies. The quantification of HA-NbATG3 protein accumulation is presented. The results are representative of three independent experiments

Since GAPCs interact with ATG3 to negatively regulate autophagy in plants (Han et al. 2015), we further examined whether RSV CP affected the interaction between GAPC and ATG3. The interaction between RSV CP and NbATG3 was first examined by using a BiFC assay. The results showed that CP could not bind to ATG3 (Supplementary Fig. 2B). The BiFC assay was used to observe the interaction of NbATG3 and NbGAPC2 in the presence of RSV CP. We co-expressed HA-tagged CP or Luc (as a control) with BiFC combination plasmids (ATG3-cYFP and NbGAPC2-nYFP) in *N. benthamiana.* The expression of NbGAPC2-nYFP and NbATG3-cYFP was confirmed by using Western blot assay (Fig. 5C) Interestingly, the intensity of reconstituted fluorescence was markedly reduced in the presence of wild-type CP compared with the CP_N4A_ mutant or Luc-HA (Fig. 5A and B). We further verified that RSV CP interfered with the interaction between NbATG3 and NbGAPC2 via a competitive pull-down assay. The fusion proteins of MBP-NbATG3, NbGAPC2-His, and MBP-CP were prepared from *E. coli* BL21 (DE3). The fusion proteins MBP-NbATG3 and NbGAPC2-His were mixed with the competitive proteins MBP and MBP-CP with serially reduced and increased amount, respectively (Fig. 5D). NbGAPC2-His and its interactors were pulled down by using Ni–NTA agarose for His-tagged proteins and then subjected to western blot analysis. The results showed that the amount of co-pulled down MBP-NbATG3 was reduced in accordance with the increased amount of competitive MBP-CP (Fig. 5D). Furthermore, MBP-CP or MBP-CP_N4A_ were incubated with fusion proteins GST-NbATG3 and NbGAPC2-His and pulled down using Ni–NTA agarose. The resulting eluates were examined by western blot analysis (Fig. 5E). The results showed that the amount of GST-NbATG3 bound to NbGAPC2-His was reduced in the presence of MBP-CP but not the MBP-CP_N4A_ or unfused MBP. Together these results suggested that the interaction of RSV CP with NbGAPC2 disrupts the interaction of NbGAPC2 and NbATG3 in vitro.

To confirm the competition between CP and NbATG3 to interact with NbGAPC2, we again expressed NbGAPC2 tagged with GFP and ATG3 tagged with HA in *N. benthamiana* plants and incubated them with MBP or MBP-CP fusion proteins and GFP-Trap beads. The immunoprecipitates were detected using GFP, HA, and MBP antibodies. The results revealed that less HA-ATG3 was co-precipitated in the presence of wild-type CP (MBP-CP) compared with the CP_N4A_ mutant (MBP-CP_N4A_) or unfused MBP (Fig. 5F). These results agreed well with those of the competitive pull-down assay (Fig. 5D). Together, the pull-down assay and co-IP analysis demonstrated that CP disrupted the interaction of NbATG3 and NbGAPC2 by competitively binding to NbGAPC2. The CP_N4A_ mutant failed to bind to NbGAPC2; therefore, these results suggested that CP induces autophagy by interfering with the interaction of GAPC2 and ATG3.

### RSV CP upregulates the transcription of NbATG3

**Fig. 6:**
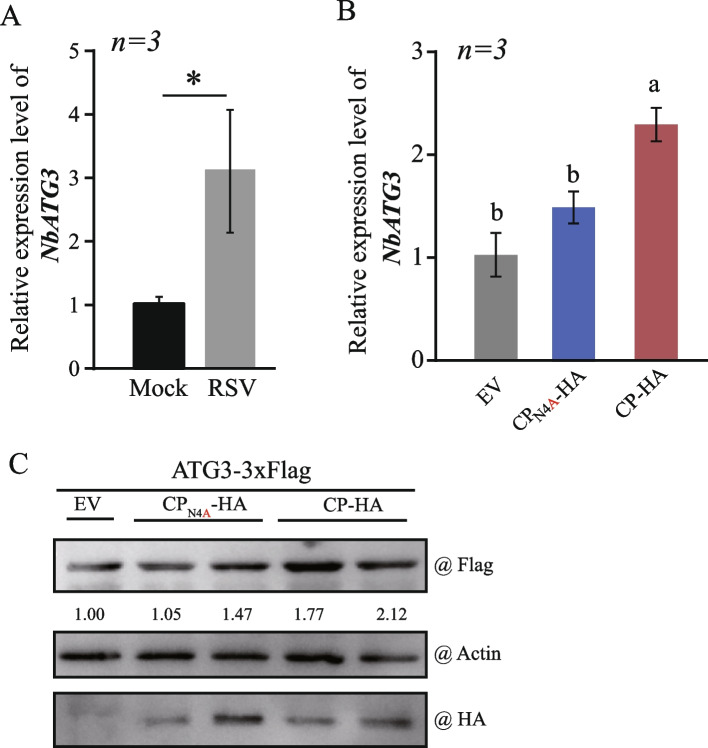
The expression of the *NbATG3 *is upregulated by RSV or RSV CP. **A **and **B **RT-qPCR showed the upregulation of NbATG3 in RSV infection (A) or transiently expressed CP and CP_N4A_-HA(B) in *N. benthamiana. *The error bars represent the mean from three biological replicates. “*” indicates *P *< 0.05 (Student’s *t*-test) (A). Different letters above the bars indicate significant differences (*P* < 0.05, one-way ANOVA) (B). **C **NbATG3-3XFlag was co-expressed with CP-HA and CP_N4A_-HA, and its expression was examined by western blot with an anti-Flag antibody. The accumulation of NbATG3-3XFlag protein was quantified by ImageJ and normalized to actin. The number indicates the relative NbATG3-3XFlag accumulation compared to the control sample (EV), which was set to 1.00. **C **The results are representative of three independent experiments

The increased accumulation of ATG3 enhances autophagic activity (Han et al. 2015). Therefore, we tested whether RSV infection or the over-expression of CP could enhance the expression of ATG3 in *N. benthamiana* plants. The transcription levels of NbATG3 were examined using qRT-PCR during RSV infection or transiently expressed RSV CP alone. As shown in Fig. 6, the transcription level of NbATG3 was upregulated both in RSV-infected plants (7 dpi; Fig. 6A) and in the presence of RSV CP expression (3 dpi; Fig. 6B), whereas the CP_N4A_ mutant did not significantly increase the expression of NbATG3 (Fig. 6B). We then used a native NbATG3 promoter to express NbATG3 tagged with 3xFlag in *N. benthamiana* plants. Total proteins were extracted and NbATG3-3xFlag accumulation was examined by using the anti-Flag antibody. The results showed that the amount of NbATG3 was remarkably increased (1.71 to 2.12 fold) when co-expressed with RSV CP (Fig. 6C). In comparison, the level of enhancement of NbATG3 expression was lower in the presence of the CP_N4A_ mutant than in the presence of wild-type CP (Fig. 6C). These results indicated that RSV CP enhances the expression of NbATG3 by regulating NbATG3 promoter activity. However, the reason CP elevates the NbATG3 expression is required more studies in the future.

### RSV CP inhibits the autophagic degradation of RSV MP and NS3

**Fig. 7:**
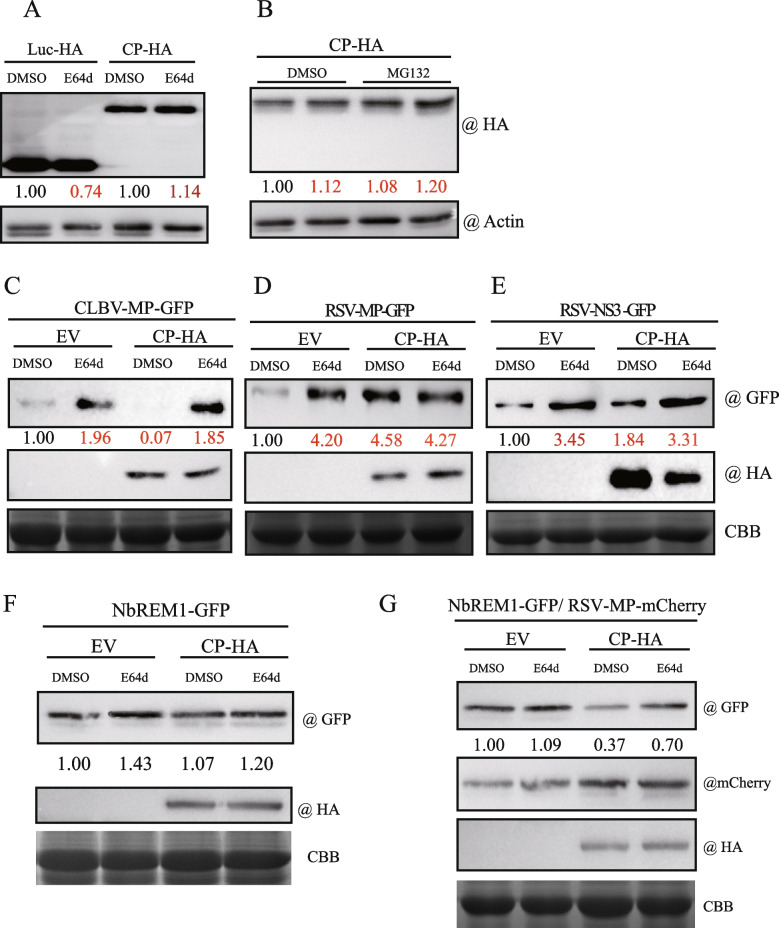
Effects of RSV CP on protein autophagic degradation. **A **and **B **CP-HA was expressed in *N. benthamiana *leaves and treated with 100 μM E64d, an autophagy inhibitor (A), or 100 μM MG132, a proteasome inhibitor (B). The protein accumulation was examined by western blot with an anti-HA antibody. The quantification of protein was performed by ImageJ and normalized to actin. **C-E **CP-HA was co-expressed with the viral proteins CLBV-MP (C), RSV-MP (D), or RSV-NS3 (E) with GFP tags in *N. benthamiana *and treated with 100 μM E64d. **F **and **G **CP-HA was co-expressed with the host protein NbREM1-GFP in the absence (F) or presence (G) of RSV-MP tagged with mCherry in *N. benthamiana *and treated with 100 μM E64d. The total protein was extracted and detected by western blot with anti-GFP, anti-mCherry, and anti-HA antibodies. Coomassie blue-stained proteins are shown as the loading control. The quantification of protein was performed by ImageJ and normalized to actin or the loading control. The number indicated the relative protein accumulation compared to the control sample (Luc-HA or EV), which was set to 1.00. The results are representative of three independent experiments

Autophagy has been shown to suppress RSV infection. Silencing the autophagy-related genes ATG5 and ATG7 in *N. benthamiana* can promote RSV accumulation, suggesting that autophagy acts as an antiviral defense against RSV multiplication (Jiang et al. 2021). Furthermore, other studies revealed that RSV-encoded MP and the silencing suppressor protein (NS3) were targeted for autophagic degradation in host plants (Fu et al. 2018; Jiang et al. 2021). Our study demonstrated that RSV CP alone could promote autophagic activity in *N. benthamiana* (Figs. 1E and 4). Thus, it is interest to determine whether RSV CP-activated autophagy can elevate the autophagic degradation of viral proteins. First, we tested the stability of RSV CP in the cell. Apart from the autophagy pathway, the ubiquitin–proteasome pathway is another protein degradation pathway in eukaryotes (Wang 2015). We transiently expressed CP tagged with HA at the C-terminal in *N. benthamiana* and treated it with the autophagy inhibitor E64d and proteasome inhibitor MG132. The accumulation of RSV CP-HA with or without treatment was analyzed by western blot (Fig. 7A and B). The results showed that RSV CP-HA was relatively stable in the plants and not degraded by the autophagy and proteasome pathways.

Viral proteins, such as MP encoded by CLBV, MP and NS3 encoded by RSV were previously reported to be degraded through the autophagy pathway (Niu et al. 2021; Li et al. 2021; Jiang et al. 2021). As RSV CP elevates autophagic activity, the autophagic degradation of viral proteins might be promoted by the presence of RSV CP. To investigate this hypothesis, CLBV MP, RSV MP, and RSV NS3 were fused to GFP at the C-terminal and co-expressed with RSV CP along with treatment with the autophagy inhibitor E64d. The extent of protein degradation was evaluated by the ratio of the accumulation of viral protein (normalized to total protein) to that without E64d treatment in the absence of RSV CP. The results showed that in the presence of RSV CP, the accumulation level of CLBV MP was significantly reduced to a ratio of 0.07. With E64d treatment, in the absence and presence of RSV CP, the accumulation level of CLBV MP was about 1.96 and 1.85, respectively, suggesting reduced CLBV MP abundance due to autophagic degradation (Fig. 7C). The accumulation of RSV MP and RSV NS3 in the absence of RSV CP was remarkably lower than their accumulation with E64d treatment, indicating that they were readily targeted and degraded by autophagy without induction by RSV CP (Fig. 7D and E). However, different from CLBV MP, RSV MP and RSV NS3 accumulation was elevated when co-expressed with RSV CP, showing that they were stabilized in the presence of RSV CP (Fig 7D and E).

### RSV CP induces the autophagic degradation of NbREM1

RSV MP facilitates the autophagic degradation of NbREM1 by interfering with the S-acylation modification of NbREM1 (Fu et al. 2018). To examine the effects of RSV CP on NbREM1 stability, we transiently co-expressed NbREM1-GFP and CP-HA along with E64d treatment. Western blot analysis showed that NbREM1-GFP accumulation was similar with or without CP expression (Fig. 7F), suggesting that RSV CP expression alone does not induce NbREM1 autophagic degradation. Interestingly, when RSV MP-mCherry was included in the same co-expression experiment, NbREM1-GFP accumulation was markedly lower in the presence of CP-HA than in its absence without E64d treatment (Fig. 7G), suggesting that after the interruption of the S-acylation modification of NbREM1 by RSV MP, RSV CP expression can promote the autophagic degradation of NbREM1.

## Discussion

Numerous studies have showed that autophagy plays a general role in antiviral defenses, however autophagy is also utilized by some viruses to facilitate their infection. In the case of RSV, the viral proteins MP and NS3, which are involved in viral movement and the suppression of antiviral RNA silencing activity, are targeted and degraded by the autophagy pathway to limit viral multiplication (Fu et al. 2018; Jiang et al. 2021). In contrast, RSV MP induces autophagic degradation of NbREM1 to overcome the inhibition of viral movement (Fu et al. 2018). Interestingly, our study demonstrated that RSV CP plays the dual functions of elevating the autophagic degradation of host antiviral protein (Fig. 7G) and protecting RSV proteins against autophagic degradation (Fig. 7D and E). Silencing of GAPC reduced RSV accumulation, while silencing of ATG5 and ATG7 promoted RSV accumulation (Jiang et al. 2021 and Supplementary Fig. 3). These observations indicate that autophagy-mediated antiviral responses still play a significant part in inhibition of RSV accumulation in plants, although RSV utilizes autophagy to facilitate its infection and also has evolved the strategy to protects its proteins against autophagic degradation. Hence, autophagy acts as a double-edged sword and plays dual contrasting roles in the interaction between RSV and host plants during infection.

The RSV CP is essential for viral biological processes as it is involved in the formation of RNP particles, viral transcription, and replication (Hong et al. 1991). It is generally established that aside from its role in enclosing the viral genome, the viral CP is commonly associated with other processes in the viral infection cycle. The diverse functions of CP are implemented via interactions with viral and host components during viral infection (Lan 2016). As a major component of RNPs, RSV CP is the most likely to be recognized for vector transmission. Five proteins, atlasin, novel cuticle protein, jagunal, nascent polypeptide-associated complex domain protein, and vitellogenin, were identified as interacting with RSV CP in the insect hemolymph (Kormelink et al. 2021). RSV CP was also found to interact with a 60S ribosomal protein L18 (Shuo et al., 2018) and G protein pathway suppressor 2 (Li et al. 2019) to support viral replication and translation in host cells*.* In addition, RSV CP (pc3) can self-interact (Kim et al. 2014) and binds to the RSV non-structural MP (NSvc4) to promote viral RNP transmission and accumulation in cells of the insect *Laodelphax striatellus* (Wu et al. 2014). RSV CP also induces the jasmonic acid (JA) pathway to attract insect vectors for effective viral transmission (Han et al. 2020). Thus, RSV CP is a multifaceted protein in the virus-host interaction.

Here, we found that RSV CP could enhance autophagic activity via specific interaction with GAPCs, which are negative regulators of autophagy (Fig. 2). GAPCs negatively regulate autophagy by binding to ATG3 (Han et al. 2015). RSV CP out-competed ATG3, resulting in the disruption of GAPC-ATG3 binding and leading to a higher level of autophagic activity (Fig. 4). Besides being an autophagy key factor, ATG3 acts as a regulator of plant immunity-related cell death (Liu et al. 2005). It is worth noting that the βC1 protein, encoded by CLCuMuB, elevates autophagy by disrupting the interactions of GAPCs and ATG3 (Ismayil et al. 2020). On the other hand, CP encoded by CWMV interacts with GAPCs to promote the interaction between GAPC2 and ATG3, leading to the inhibition of autophagy (Niu et al. 2022). Together, these studies suggest that plant viruses commonly target GAPCs and ATG3 to modulate autophagy in plants.

Several studies have implicated GAPCs in plant resistance. The silencing of GAPCs can promote autophagic activity (Han et al. 2015). The downregulation of GAPCs in *N. benthamiana* reduced the accumulation of CWMV, bamboo mosaic virus (BaMV), and satBaMV RNA (Prasanth et al. 2011; Niu et al. 2022). In addition to its roles as autophagy regulators, GAPCs are essential for maintaining cellular ATP levels and carbohydrate metabolism by catalyzing the critical reaction of glycolysis (Cerff 1978; Guo et al. 2014). Viruses have developed numerous strategies to hijack cellular metabolism and biosynthesis machinery for their specific needs. For example, viral glycoprotein 5 (GP5) interacts with GAPC to facilitate the replication of porcine reproductive and respiratory syndrome virus (PRRSV) via its glycolytic activity (Liu et al. 2021). Tomato bushy stunt virus (TBSV) regulates cellular metabolic pathways by co-opting aerobic glycolytic enzymes to produce ATP molecules within the replication compartment and enhance virus production (Nagy and Lin 2020). TBSV recruits the glycolytic NADH-producing GAPC into viral replicase complexes to take advantage of GAPC as an RNA chaperone during ( +) RNA synthesis (Huang and Nagy 2011; Wang and Nagy 2008). Plants have two glycolytic pathways separately located in the cytosol and plastid (Plaxton 1996; Schwender et al. 2003). *N. benthamiana* and *O. sativa* contains three GAPCs in the cytosol. We found that all NbGAPCs (NbGAPC1, NbGAPC2, and NbGAPC3) and two OsGAPCs (OsGAPC2 and OsGAPC3) interacted with RSV CP (Fig. 2), implying that the interaction with GAPCs is important for viral infection. Whether RSV CP recruits GAPCs to regulate or hijack the glycolytic metabolic pathway of the host to promote viral replication remains unknown and needs to be investigated further. It was observed that stress conditions can induce the translocation of GAPC to the nucleus (Kim et al. 2022). It is also interesting to further examine whether RSV CP affects subcellular localization and enzymatic activities of GAPC and the possible consequence in the course of RSV infection.

We found that RSV CP could elevate autophagy in plants (Figs. 1 and 4); however, it was stable against autophagy and proteasomal degradation (Fig. 7A and B). In contrast, the accumulation of MP and NS3 was increased with E64d treatment (Fig. 7D and E), suggesting that MP and NS3 were subjected to selective autophagic degradation. The plant protein NbP3IP was previously identified as guiding the autophagic degradation of NS3 (Jiang et al. 2021). Interestingly, the degradation of MP and NS3 was inhibited by RSV CP expression (Fig. 7D and E). The CaMV gene VI product (P6), a major component of viral factory inclusion, protects P4 against autophagic degradation by sequestering it and coordinating particle assembly and storage (Hafrén et al. 2017). RSV MP was reported to directly bind to RSV CP (Zhang et al. 2008). Whether RSV CP protects RSV MP and NS3 through direct interaction is unclear. The mechanism by which RSV CP suppresses the autophagic degradation of other RSV proteins needs to be investigated further.

We also found that RSV CP-triggered autophagy could promote the autophagic degradation of NbREM1 (Fig. 7G), suggesting that RSV employs autophagy to benefit viral infection. Furthermore, RSV CP-triggered autophagy could elevate the degradation of an unrelated viral protein (MP) encoded by CLBV (Fig. 7C). The molecular mechanism by which RSV CP induces selective autophagy to target antiviral components but not its own viral products remain unclear and warrants further investigation.

## Materials and methods

### Plant materials and virus inoculation

*N. benthamiana* plants were grown in a greenhouse at 25 °C, with 70% relative humidity and 16 h of daylight. RSV was obtained from Nanjing City, Jiangsu province, China, kindly provided by Dr. Tong Zhou (Jiangsu Academy of Agricultural Sciences). The mechanical inoculation of RSV on *N. benthamiana* plants was performed as described previously (Kong et al. 2014). Briefly, the leaves of *N. benthamiana* plants at the six-leaf stage were dusted with carborundum powder and mechanically rubbed with viral inoculum prepared from RSV-infected rice leaves in 20 mM sodium phosphate buffer at pH 7.0.

### Plasmid constructs

Total RNA was extracted from RSV-infected *N. benthamiana* leaves using Trizol (Invitrogen) and supplied as a template for reverse transcription (RT) using ReverTra Ace reverse transcriptase (Toyobo, Japan). The DNA fragments of RSV genes were amplified by PCR (polymerase chain reaction) using PrimeSTAR® HS DNA Polymerase (Takara Bio) and cloned into the responsive plasmid using the ClonExpress II One Step Cloning Kit (Vazyme, Nanjing, China). All plasmid constructs generated in this study are described in Supplementary Table 1. The Flag peptide was added at the N-terminal of ATG3. GFP-ATG8f has been described previously (Niu et al. 2022). All primers used in this study are listed in Supplementary Table 2.

### *Agrobacterium* infiltration

The plasmid constructs were transformed into *Agrobacterium tumefaciens* strain GV3101. Agroinfiltration was performed as described previously.

### Co-immunoprecipitation and mass spectrometry analysis

The RSV CP-GFP protein was transiently expressed and extracted from *N. benthamiana.* The GFP protein was prepared in parallel as a control. Co-immunoprecipitation (Co-IP) assays using GFP-Trap beads (ChromoTek, Germany) were performed as described previously (Sun et al. 2006). LC–MS/MS (Liquid Chromatograph Mass Spectrometer) and bioinformatics analyses for protein identification were performed by Shanghai Applied Protein Technology Co., Ltd.

### Fluorescent protein expression and visual observation

The GFP-tagged and bimolecular fluorescence complementation (BiFC) proteins were transiently co-expressed on 4-week-old *N. benthamiana* leaves. At 3 dpi, the fluorescent proteins were observed using an FV3000 confocal microscope (Olympus, Japan). The fluorescence signals were visualized with laser excitation/emission filters of 488/500–510 nm for GFP and 514/580–600 nm for YFP (BiFC).

### Western blot analysis

Western blot analysis was performed as described previously (Niu et al. 2022). Anti-GFP (1:5000, Sigma, Cat. No. F1804), anti-Flag (1:5000, EASYBIO, Cat. No. BE7001), anti-HA, anti-His, and anti-maltose-binding protein (MBP; all 1:5000, Beijing Protein Innovation Co., Ltd.) antibodies, as well as secondary goat anti-mouse immunoglobulin G-horseradish peroxidase (IgG-HRP; 1:10,000, Proteintech) were used for the detection of GFP-, HA-, Flag-, His-, GST-, and MBP-tagged proteins. The actin protein was detected using a primary anti-actin antibody (1:5000, Kangwei). The detection of ATG8 and phosphatidylethanolamine-conjugated ATG8 (ATG8-PE) was carried out as described previously (Niu et al. 2022) using an anti-ATG8 primary antibody (1:2000, Abcam).

### Competitive pull-down assay

The fusion proteins of MBP-ATG3, MBP-CP, GST-ATG3, and NbGAPC2-His were expressed in the *Escherichia coli* strain BL21 and purified using Maltose-Binding Glutathione Sepharose TM 4 Fast Flow (GE Healthcare) or Ni–NTA agarose (Qiagen) according to the manufacturer’s instructions. His pull-down assays were performed as described previously (Sun et al. 2013). Finally, samples were analyzed by western blotting with anti-GST (1:5000), anti-His (1:5000), and anti-MBP (1: 5000) antibodies.

### Competitive Co-IP assay

The competitive protein RSV CP was fused with MBP and expressed in *E. coli* BL21 (DE3). The MBP-CP, MBP-CP_N4A_, and MBP proteins were purified using amylose resin with gradient column buffer as described previously (Sun et al. 2013). NbGAPC2-GFP and HA-ATG3 were transiently co-expressed in *N. benthamiana* leaves. The total proteins containing NbGAPC2-GFP and HA-ATG3 proteins were extracted from *N. benthamiana* leaves and mixed well with the purified MBP or MBP-CP (40 μg/ml), respectively, and subjected to immunoprecipitation using 20 μL GFP-Trap beads (ChromoTek, Germany) as described previously. Precipitates were washed five times with a wash buffer. Finally, samples were analyzed by western blotting with anti-GFP (1:5000), anti-HA (1:5000), and anti-MBP (1: 5000) antibodies.

### Transmission electron microscope (TEM)

*N. benthamiana* leaf tissues with or without RSV infection (14 dpi) were prepared, subjected to vacuum infiltration, and fixed immediately with 2.5% glutaraldehyde (Sigma, G5882) in 0.1 M PBS overnight at 4 °C. Samples were washed three times with PBS, post-fixed with 1% OsO_4_ (Sigma, O5500), rinsed three times with PBS again, dehydrated in a graded ethanol series followed by the replacement of ethanol with acetone, and embedded in SPI-PON812 resin (SPI Science, 90,529–77–4). The ultrathin sections were stained with 2% (w/v) uranyl acetate (Polysciences, 21,447–25) and 2.6% (w/v) lead citrate (Sigma, 15,326). Images were observed and captured using a transmission electron microscope (TEM; Hitachi H-7650, Japan) at 80 kV (Guan et al. 2022).

## Supplementary Information

**Additional file 1: Supplementary Fig 1. **Co-immunoprecipitation of RSV CP with proteins obtained from N.benthamiana and analyzed by mass spectrometry (IP-MSMS). **Supplementary Fig 2.** BiFC assay analyzed the protein interaction in N. benthamianabetween CP_N4A_ and OsGAPC2, CP and NbATG3. **Supplementary Fig 3.** RSV symptoms and accumulation in transgenic plants of NbGAPC2-RNAi after virus inoculation. **Supplementary Table 1.** List of plasmid constructs generated in this study. **Supplementary Table S2. **A list of primers used in this study.

## Data Availability

Data are available from corresponding author upon reasonable request.
