# Supplementary material for: Coat protein of rice stripe virus enhances autophagy activity through interaction with cytosolic glyceraldehyde-3-phosphate dehydrogenases, a negative regulator of plant autophagy
Source: Stress Biol. 2023 Mar 23;3(1):3. doi: 10.1007/s44154-023-00084-3 (PMC10441990; doi:10.1007/s44154-023-00084-3)
Supplement: Supplementary file 1 — Additional file 1: Supplementary Fig 1. Co-immunoprecipitation of RSV CP with proteins obtained from N.benthamiana and analyzed by mass spectrometry (IP-MSMS). Supplementary Fig 2. BiFC assay analyzed the protein interaction in N. benthamianabetween CPN4A and OsGAPC2, CP and NbATG3. Supplementary Fig 3. RSV symptoms and accumulation in transgenic plants of NbGAPC2-RNAi after virus inoculation. Supplementary Table 1. List of plasmid constructs generated in this study. Supplementary Table S2. A list of primers used in this study. [file 44154_2023_84_MOESM1_ESM.pdf]

A

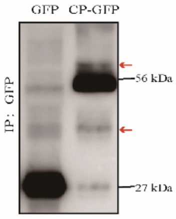

B

Table 1 Screened host candidate genes with CP-GFP bait in *N. benthamiana*

Gene ID	Gene name	
Niben101Scf02502g16008.1	*PK1*	Pyruvate kinase family protein
Niben101Scf19612g01003.1	G6PDH1	Glucose-6-phosphate dehydrogenase 1
Niben101Scf01066g04002.1	*HAD*	HAD-superfamily hydrolaseHAD
Niben101Scf03133g00003.1	*GAPC2*	Glyceraldehyde-3-phosphate dehydrogenase
Niben101Scf05438g05014.1	*AMT*	Aminomethyltransferase
Niben101Scf04444g06007.1	*FAS1*	Fasciclin-like arabinogalactan protein 1
Niben101Scf07103g01015.1	*ATP-β*	ATP synthase subunit beta

Supplementary Fig 1. Co-immunoprecipitation of RSV CP with proteins obtained from *N. benthamiana* and analyzed by mass spectrometry (IP-MSMS). Total proteins were extracted from *N. benthamiana* leaves expressing CP-GFP as a bait protein and incubated with covalently coupled GFP beads. The sample containing non-fused GFP protein was used in parallel as a control. (A) The proteins were eluted and separated via SDS-PAGE. The selected protein bands in the CP-GFP samples are indicated by arrowheads. (B) The candidate proteins obtained from IP-MSMS are listed.

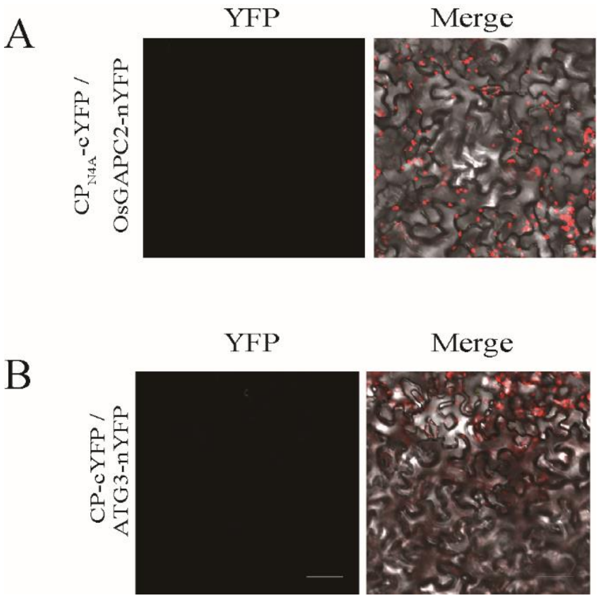

Supplementary Fig 2. BiFC assay analyzed the protein interaction in *N. benthamiana* between CP_{N4A} and OsGAPC2, CP and NbATG3

BiFC assay analyzed the protein interaction in *N. benthamiana.* (A) The CP_{N4A} were fused to the C-terminal portion of YFP (cYFP) and OsGAPC2 fused to the N-terminal portion of YFP (nYFP), The YFP fluorescence in epidermal cells was observed by confocal laser scanning microscopy. (B) NbATG3 were fused to the N-terminal portion of YFP (nYFP). The recombinant plasmids of CP with NbATG3 were co-expressed in *N. benthamiana* (B). No YFP fluorescence indicated no protein interactions. The merged confocal images show YFP fluorescence, bright field, and chloroplast auto-fluorescence overlapping. Scale bars, 20 μm.

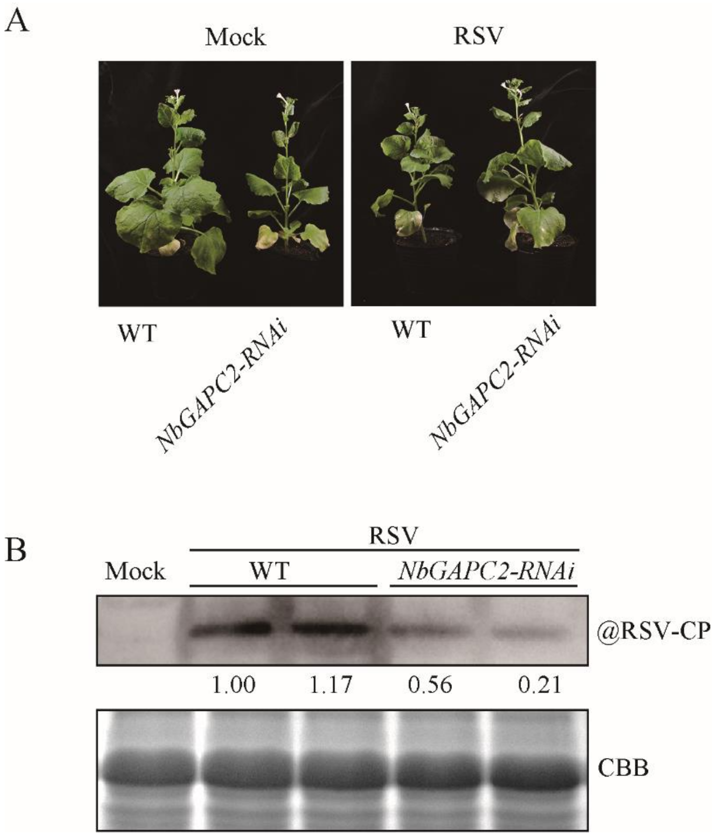

Supplementary Fig 3. RSV symptoms and accumulation in transgenic plants of *NbGAPC2-RNAi* after virus inoculation.

(A) RSV symptoms in transgenic plants of *NbGAPC2-RNAi* for 25 days after virus inoculation.

(B) CP protein accumulation of RSV in transgenic plants of *NbGAPC2-RNAi* after virus inoculation.

Supplementary Table 1. List of plasmid constructs generated in this study.

**Construct**	**Insertion (region)**[a]	**Plasmid**[b]	**Restriction sites**
CP-HA	RSV CP	pCAMBIA-1302-GFP	*Spe*I
CP_{N4A}-HA	RSV CP	pCAMBIA-1302-GFP	*Spe*I
NbGAPC2-GFP	NbGAPC2	pCAMBIA-1302-GFP	*Spe*I
OsGAPC2-GFP	OsGAPC2	pCAMBIA-1302-GFP	*Spe*I
CP-cYFP	RSV CP	pCAMBIA1302-cYFP	*KpnI BamHI*
NbGAPC1-nYFP	NbGAPC1	pCAMBIA1302-nYFP	*KpnI BamHI*
NbGAPC2-nYFP	NbGAPC2	pCAMBIA1302-nYFP	*KpnI BamHI*
NbGAPC3-nYFP	NbGAPC3	pCAMBIA1302-nYFP	*KpnI BamHI*
OsGAPC2-nYFP	OsGAPC2	pCAMBIA1302-nYFP	*KpnI BamHI*
CPΔ59-322-cYFP	RSV CPΔ59-322	pCAMBIA1302-nYFP	*KpnI BamHI*
CPΔ58-cYFP	RSV CPΔ58	pCAMBIA1302-nYFP	*KpnI BamHI*
CPΔ4-cYFP	RSV CPΔ4	pCAMBIA1302-nYFP	*KpnI BamHI*
CPΔ3-cYFP	RSV CPΔ3	pCAMBIA1302-nYFP	*KpnI BamHI*
CP $_{N4A}$-cYFP	RSV CP N4A	pCAMBIA1302-nYFP	*KpnI BamHI*
NbATG3-cYFP	NbATG3	pCAMBIA1302-cYFP	*KpnI BamHI*
NbATG3-nYFP	NbATG3	pCAMBIA1302-nYFP	*KpnI BamHI*
MBP-CP	RSV CP	pMAL-C2X	*XbaI HindIII*
MBP-CP_{N4A}	RSV CP_{N4A}	pMAL-C2X	*XbaI HindIII*
GST-NbATG3	NbATG3	pGEX-4T-1	*BamHI Spe*I
NbGAPC2-His	NbGAPC2	pET28a	*BamHI*
MBP-ATG3	NbATG3	pMAL-C2X	*XbaI HindIII*
MP- mCherry	RSV-MP	pBin121- mCherry	*BamHI*
NbREM1.1-GFP	NbREM1.1	pCAMBIA-1302-GFP	*Spe*I
HA-NbATG3	NbATG3	pCAMBIA-1302-GFP	*Spe*I
CLBV-MP-GFP	CLBV-MP	pCAMBIA-1302-GFP	*Spe*I
NS3-GFP	RSV NS3	pCAMBIA-1302-GFP	*Spe*I
MP-GFP	RSV MP	pCAMBIA-1302-GFP	*Spe*I
GFP-NbATG8f	NbATG8f	pBin61-GFP	*BamH*I
Flag-NbATG3	NbATG3	pTCK303-Flag	*BamH*I *Spe*I
Luc-HA	Luc	pCAMBIA-1302	*Spe*I
Luc-Flag	Luc	pTCK303-Flag	*BamH*I *Spe*I
CP-AD	RSVCP	pGADT7	*EcoRI XhoI*
NbGAPC2-BD	NbGAPC2	pGBKT7	*EcoRI BamH*I
OsGAPC2-BD	OsGAPC2	pGBKT7	*EcoRI BamH*I

[a]Accession number:

CP (GU230170.1), NbGAPC1(XM_019383154), NbGAPC2 (KM986324), NbGAPC3 (KM986325), OsGAPC1(NP_001390311.1), OsGAPC2 (NP_001389310.1), OsGAPC3(NP_001388829.1), RSV-NS3 (EF493242.1), RSV-MP (AY973301.1), NbATG3 (KX369396), NbATG8f (KU561372), CLBV-MP (MH339992.1), NbREM1.1(ON392760.1).

[b]Plasmid references:

pCambia1302-GFP (Zhang et al., 2011), pBin61-GFP (Voinnet et al., 2000); pTCK303 (Li et al., 2011)

Li, W., Han, Y., Tao, F., and Chong, K. (2011). Knockdown of SAMS genes encoding S-adenosyl-l-methionine synthetases causes methylation alterations of DNAs and histones and leads to late flowering in rice. Journal of plant physiology **168**:1837-1843.

Sun, L., Andika, I.B., Kondo, H., and Chen, J. (2013). Identification of the amino acid residues and domains in the cysteine-rich protein of C hinese wheat mosaic virus that are important for RNA silencing suppression and subcellular localization. Molecular plant pathology **14**:265-278.

Voinnet, O., Lederer, C., and Baulcombe, D.C. (2000). A viral movement protein prevents spread of the gene silencing signal in Nicotiana benthamiana. Cell **103**:157-167..

Zhang, Y., Zhang, X., Niu, S., Han, C., Yu, J., and Li, D. (2011). Nuclear localization of Beet black scorch virus capsid protein and its interaction with importin α. Virus research **155**:307-315.

Supplementary Table S2. A list of primers used in this study.

Clone Name	Primer Name	Oligonucleotide sequence (5'- 3')
**Primers used for transient expression in *N. benthamiana***		
pCambia1302-CP-HA	F_{speI}-CP	accatggtagatctgactagtATGGGTACCAACAAGC
	R_{speI}-CP-HA	gctcaccatcctaggactagtTCAAGCGTAATCTGGAACATCGTATGGGTAGTCATCTGCACCTTCTG
pCambia1302- CP_{N4A}-HA	F_{speI}- CP_{N4A}-HA	accatggtagatctgactagtATGGGTACCGCCAAGC
	R_{speI}- CP_{N4A}-HA	gctcaccatcctaggactagtTCAAGCGTAATCTGGAACATCGTATGGGTAGTCATCTGCACCTTCTG
pCambia1302-NbGAPC2-GFP	F_{speI}- NbGAPC2-GFP	accatggtagatctgactagtATGGCCAAGGTTAAGATT
	R_{speI}- NbGAPC2-GFP	gctcaccatcctaggactagtCTGAACCGATGCCATGT
pCambia1302- OsGAPC2-GFP	F_{speI}- OsGAPC2-GFP	accatggtagatctgactagtATGGCGAAGATTAAG
	R_{speI}-OsGAPC2-GFP	gctcaccatcctaggactagtCTGGGTGTTGTACATGTG

pCambia1302- NbGAPC1-nYFP	F_{KpnI}-NbGAPC1	gaattctgcagtcgacggtaccATGGCATCTGACAAG
	R_{BamHI}-NbGAPC1	ccttgctcaccatcaggatccAGCAACAGAAGCCATATG
pCambia1302- CP-cYFP	F_{KpnI}-CP	gaattctgcagtcgacggtaccATGGGTACCAACAAGC
	R_{BamHI}-CP	gctgcacgctgcccaggatccGTCATCTGCACCTTCT
pCambia1302-NbGAPC2-nYFP	F_{KpnI}-NbGAPC2	gaattctgcagtcgacggtaccATGGCCAAGGTTAAGATT
	R_{BamHI}-NbGAPC2	ccttgctcaccatcaggatccCTGAACCGATGCCATGT
pCambia1302-NbGAPC3-nYFP	F_{KpnI}-NbGAPC3	gaattctgcagtcgacggtaccATGGCCAAGGTTAAG
	R_{BamHI}-NbGAPC3	ccttgctcaccatcaggatccTTACTGGACTGATGCCATGTGC
pCambia1302-OsGAPC2-nYFP	F_{KpnI}-OsGAPC2	gaattctgcagtcgacggtaccATGGCGAAGATTAAGATC
	R_{BamHI}- OsGAPC2	ccttgctcaccatcaggatccCTGGGTGTTGTACATG
pCambia1302-CPΔ59-322-cYFP	F_{KpnI}- CPΔ59-322	gaattctgcagtcgacggtaccATGGGTACCAACAAGC
	R_{BamHI}- CPΔ59-322	gctgcacgctgcccaggatccTTTGTCCTTCAATATGCC

pCambia1302-CPΔ58-cYFP	F_{KpnI} - CPΔ58	gaattctgcagtcgacggtaccATGGGTGGTGACACACTGGCC
	R_{BamHI}- CPΔ58	gctgcacgctgcccaggatccGTCATCTGCACCTTCT
pCambia1302-CPΔ4-cYFP	F_{KpnI} – CPΔ4	gaattctgcagtcgacggtaccATGAAGCCAGCCACTCTAGC
	R_{BamHI}– CPΔ4	gctgcacgctgcccaggatccGTCATCTGCACCTTCT
pCambia1302-CPΔ3-cYFP	F_{KpnI} – CPΔ3	gaattctgcagtcgacggtaccATGAACAAGCCAGCCACTC
	R_{BamHI}– CPΔ3	gctgcacgctgcccaggatccGTCATCTGCACCTTCT
pCambia1302- CP_{N4A}-cYFP	F_{KpnI} –CP_{N4A}	gaattctgcagtcgacggtaccATGAACAAGCCAGCCACTC
	R_{BamHI}–CP_{N4A}	gctgcacgctgcccaggatccGTCATCTGCACCTTCT
pCambia1302-NbATG3-cYFP	F_{KpnI} - NbATG3	gaattctgcagtcgacggtaccATGGTACTGTCGCAGAAG
	R_{BamHI}- NbATG3	gctgcacgctgcccaggatccGGTGCTGCTGCTACC
pCambia1302-NbATG3-nYFP	F_{KpnI}-NbGAPC1	gaattctgcagtcgacggtaccATGGTACTGTCGCAGAAG
	R_{BamHI}-NbGAPC1-HA	ccttgctcaccatcaggatccGGTGCTGCTGCTACC

pMAL-MBP-CP	F_{XbaI}- CP	tcagaattcggatcctctagaATGGGTACCAACAAGC
	$R_{HindIII}$-CP	acgacggccagtgccaagcttGTCATCTGCACCTTCT
pMAL-MBP-CP_{N4A}	F_{XbaI}- CP	tcagaattcggatcctctagaATGGGTACCGCCAAGC
	$R_{HindIII}$-CP	acgacggccagtgccaagcttGTCATCTGCACCTTCT
pGEX-4T-1-GST-NbATG3	F $_{BamHI}$ -NbATG3	ttccaggggcccctgggatccATGGTACTGTCGCAGAAG
	R $_{EcoRI}$ -NbATG3	ctcgagtcgacccgggaattcGGTGCTGCTGCTACC
pET28a-NbGAPC2-His	F_{BamHI}- NbGAPC2	atgggtcgcggatccgaattcATGGCCAAGGTTAAGATT
	R_{SmaI}- NbGAPC2	gtggtggtggtggtgctcgagCTGAACCGATGCCATGT
pMAL-MBP-NbATG3	F_{XbaI}- NbATG3	tcagaattcggatcctctagaATGGTACTGTCGCAGAAG
	$R_{HindIII}$- NbATG3	acgacggccagtgccaagcttGGTGCTGCTGCTACCAAG
pCambia1302-HA-NbATG3	F_{speI}- NbATG3	cgccacaacatcgagggatccATGGTACTGTCGCAGAAG
	R_{speI}- NbATG3	gaattcgagctctatcccgggTCAAGCGTAATCTGGAACATCGTATGGGTAGGT

		GCTGCTGCTACC
pCambia1302-CLBV-MP-GFP	F_{speI}- CLBV-MP	accatggtagatctgactagtATGGCTTCCCTCATCAATGTGAGC
	R_{speI}- CLBV-MP	gctcaccatcctaggactagtCTTGGTCCCAGTGTCGCTGGC
pCambia1302-NS3-GFP	FspeI- NS3	accatggtagatctgactagtATGAACGTGTTCACA
	R_{speI}- NS3	gctcaccatcctaggactagtCAGCACAGCTGGA
pCambia1302-MP-GFP	F_{speI}- MP	accatggtagatctgactagtATGGCTTTGTCTCGACT
	R_{speI}- MP	gctcaccatcctaggactagtCATGATGACAGAAAC
pCambia1302-REM1.1-GFP	F_{speI}- REM1.1	accatggtagatctgactagtATGGCAGAAGTAGAAGC
	R_{speI}- REM1.1	gctcaccatcctaggactagtAAAACATCCAAGGAGTTTC
pBin121 -MP-mCherry	F_{BamHI}- MP	cacgggggactctagaggatccATGGCTTTGTCTCGACT
	R_{BamHI}- MP	tcaccatggtacccggggatccCATGATGACAGAAAC
pBin41-GFP-NbATG8f	F_{BamHI}- NbATG8f	cgccacaacatcgagggatccATGGCAAAGAGTTCATTCAAGCA

	R_{SmaI}- NbATG8f	gaattcgagctctatcccgggCACCAAGTTAAAGTCCCCAAATG
pTCK303-Flag-NbATG3	F_{BamHI}- NbATG3	cgactctagaggatccATGGTACTGTCGCAGAAG
	R_{speI}- NbATG3	gcttggcgcgactagtGGTGCTGCTGCTACCAAG
pCambia1302-Luc-HA	F_{speI}- Luc	accatggtagatctgactagtATGTCCGGTTATGTAAAC
	R_{speI}- Luc	gctcaccatcctaggactagtTCAAGCGTAATCTGGAACATCGTATGGGTA CCCACGGCGATCTTTCCG
pTCK303-Flag-Luc	F_{BamHI}- NbATG3	cgactctagaggatccATGTCCGGTTATGTAAAC
	R_{speI}- NbATG3	gcttggcgcgactagtCCCACGGCGATCTTTCCG
pGADT7-CP	F_{EcoRI}-CP	gccatggaggccagtgaattcATGGGTACCAACAAGC
	R_{BamHI}-CP	cagctcgagctcgatggatccGTCATCTGCACCTTCT
pGBKT7-NbGAPC2	F_{EcoRI}- NbGAPC2	atggccatggaggccgaattcATGGCCAAGGTTAAGATT
	R_{BamHI}- NbGAPC2	ccgctgcaggtcgacggatccCTGAACCGATGCCATGT

pGBKT7-NbGAPC2	F_{EcoRI}- NbGAPC2	atggccatggaggccgaattcATGGCCAAGGTTAAGATT
	R_{BamHI}- NbGAPC2	ccgctgcaggtcgacggatccCTGAACCGATGCCATGT
pGBKT7-OsGAPC2	F_{EcoRI}- OsGAPC2	atggccatggaggccgaattcATGGCGAAGATTAAG
	R_{BamHI}- OsGAPC2	ccgctgcaggtcgacggatccCTGGGTGTTGTACATG
**Primers used for RT-qPCR**		
	F-NbATG3-RT	ATGGTACTGTCGCAGAAGATTCACG
	R-NbATG3-RT	CCTCTTGCTAGGTTCACCTGATTCC
	F-18SrRNA-RT	GCAAGACCGAAACTCAAAGG
	R-18SrRNA-RT	TGTTCATATGTCAAGGGCTGG
